# The prevalence of pregnancy among adolescent girls and young women across the Southern African development community economic hub: A systematic review and meta-analysis

**DOI:** 10.34172/hpp.2020.51

**Published:** 2020-11-07

**Authors:** Clarence S. Yah, Sithembiso Ndlovu, Alison Kutywayo, Nicolette Naidoo, Tshepo Mahuma, Saiqa Mullick

**Affiliations:** ^1^Wits Reproductive Health and HIV Institute (Wits RHI), Faculty of Health Sciences, University of the Witwatersrand, Johannesburg, South Africa; ^2^School of Health Systems and Public Health, Faculty of Health Sciences, University of Pretoria, Pretoria, South Africa

**Keywords:** Pregnancy, Adolescents, Prevalence, Systematic review, Southern Africa

## Abstract

**Background:** Despite the high rate of HIV infections, there is still high rate of early unprotected sex, unintended pregnancy, and unsafe abortions especially among unmarried adolescent girls and young women (AGYW) 10-24 years of age in sub Saharan Africa. AGYW face challenges in accessing health care, contraception needs, and power to negotiate safer sex. This study aimed to estimate the rate of pregnancy among AGYW aged 10-24, 10-19 and 15-19 years in the Southern African Development Community (SADC) economic region.

**Methods:** A systematic review and meta-analysis was used to describe the prevalence of pregnancy among AGYW in 15 SADC member countries between January 2007 and December2017. The articles were extracted from PubMed/MEDLINE, African Index Medicus, and other reports. They were screened and reviewed according to PRISMA methodology to fulfil study eligibility criteria.

**Results:** The overall regional weighted pregnancy prevalence among AGYW 10-24 years of age was 25% (95% CI: 21% to 29%). Furthermore, sub-population 10-19 years was 22% (95% CI:19% to 26%) while 15-19 years was 24% (18% to 30%). There was a significant heterogeneity detected between the studies (I=99.78%, P < 0.001), even within individual countries.

**Conclusion:** The findings revealed a high pregnancy rate among AGYW in the SADC region. This prompts the need to explore innovative research and programs expanding and improving sexual and reproductive health communication to reduce risk and exposure of adolescents to early planned, unplanned and unwanted pregnancies, SRHR challenges, access to care, HIV/STIs, as well as other risk strategies.

## Introduction

Despite the high rate of HIV infections among adolescent girls and young women (AGYW) aged 10-24 in sub-Saharan Africa (SSA), early and/or unintended pregnancy, unsafe abortion and unprotected sex remains high in this age group.^1-13^ There are several factors associated with AGYW pregnancy in Southern Africa^14,15^; not only the pregnancy itself but the sexual and reproductive health rights (SRHR) risks and associated exposures.^12,16-19^ In addition, AGYW in sub Saharan Africa rarely access SRH care services.^2,7,9,11^ The region has high unmet contraception needs, harmful gender inequalities, high rates of sexual and gender-based violence (SGBV) and drug and alcohol abuse.^3,7,10,12,13^

Globally, over 16 million young women, aged 15 to 19 give birth each year, with more than 50% in SSA.^4,5^ The birth rate of young mothers aged 15 to 19 in SSA in 2013 was estimated at 101 births per 1000 young women.^1^ Early pregnancies have direct and indirect negative health and social consequences on young women, their families and communities,^5^ such as school dropout,^6,8^ prolonged labour, preterm birth, still births, neonatal deaths, and maternal and perinatal mortality^10-19^, particularly in SSA.^20,21^ Compared to other social public health challenges (HIV, SGBV, and youth unemployment), AGYW pregnancy interventions, awareness and information efforts are still limited.^6,22-24^ Pregnancy education and awareness varies geographically, due to differences in socio-economics, socio-cultural, and social norms.^13^

Although country specific statistics may be available, currently there is no pooled data on the rate of AGYW pregnancy and unsafe sexual practices in the SADC region^12,13,25-32^ as well as unsafe sexual practices.^13,26-29^ Therefore, our study is drawn from the fact that AGYW, aged 10-24 in Southern Africa are faced with SRHR challenges including unintended pregnancy and HIV acquisition.^12,13,25-32^ We thought pooling aggregated and disaggregated ratio describing the prevalence of pregnancy among AGYW (aged 10-24, 10-19 and 15-19) will support decision making, policy choices, policy development, evaluation, and implementation both nationally and regionally.

According to UNAIDS^33^,AGYW aged 15-24 contribute roughly 30% of all new HIV infections in Southern Africa. In South Africa alone, approximately 113 000 new HIV infections occur annually among AGYW: four times higher than their male counterparts.^34-36^ Pregnant AGYW are less likely to access early antenatal care (ANC) and test for HIV which complicates the prevention of mother to child transmission of HIV (PMTCT).^12^ Identifying and scaling up access to SRHR services aligns with universal access to health among adolescents. Hence, exploring the SRH affecting AGYW is essential for implementing interventions that can be integrated into public health policies and practices. This is particularly true for AGYW aged 10-24 who are at higher risk of HIV acquisition.^25^ Furthermore, data indicates that by the age 18 years, more than 30% of AGYW would have given birth at least once^10,25^due to high birth rates, early marriages, lack of access to health services.^13,31^ These behaviours are likely to be underpinned by unsafe sexual practices^13,29,30^ resulting in higher pregnancy rates.^23^

The purpose of this review is to estimate the pooled pregnancy among AGYW in the SADC economic region.^22,23,25-36^ The availability and accessibility of the pooled data will indicate the impact of AGYW pregnancy rate that can be used to address emerging public health challenges, both nationally and regionally within the economic region. The outcome of the study seeks to enhance cost effective interventions that link AGYW early into healthcare, empowering them to negotiate safe sex, uptake of contraception and adherence.^12,37,38^ This systematic review will therefore provide a reflection of the true pooled index pregnancy rate in the economic region which can be used as a measure in HIV and comprehensive sexuality education interventions and policy development.

## Materials and Methods

We conducted a systematic review according to the Cochrane methodology^39,40^ and used the PRISMA checklist^41^ to ensure that we applied the relevant methods. The study objectives were framed to assess the rate of pregnancy among AGYW in the 15 SADC countries: Angola, Botswana, Democratic Republic of Congo (DRC), Lesotho, Madagascar, Malawi, Mauritius, Mozambique, Namibia, Seychelles, South Africa, Swaziland, United Republic of Tanzania, Zambia and Zimbabwe. This included clear criteria for probing the literature, the search techniques/ search wordings and language, appraisal and retrieval of evidence data. The abstracts, the published articles and the data quality were reviewed according to the study eligibility criteria.

### Criteria for considering studies in this review

*Types of studies and participants*

This study focused only on AGYW pregnancy, looking for studies reporting “ever pregnant” (percentage of AGYW aged 10-24, 10-19 and 15-19 who reported ever pregnant) using a collection of search terms including “adolescent pregnancy, youth pregnancy, young women pregnancy, teen pregnancy” in SADC region.

The type of studies included in this systematic review were cross sectional, baseline data from cohort or randomized controlled trial (RCT) study designs, and from national Demographic and Health Survey (DHS) studies published as full papers, conference proceedings/abstracts, policy or reports. The eligibility screening criteria for the studies were: (1) population: all “ever pregnant” studies describing AGYW, aged between 10-24 years; (2) setting: AGYW studies from the 15 SADC economic hub countries; (3) Studies having the number of ever pregnant AGYW (n) or ever pregnant proportion (%) and 95% confidence interval (CI) of ever pregnant in addition to the total AGYW population (N). The time frame for the review was restricted from 2007 to 2017. Studies describing general population rate of pregnancy were ineligible for the study as well as studies without clear pregnancy rate among AGYW.

***Search methods for identification of studies***

*Electronic searches*

We carried out a comprehensive literature search of quantitative published studies reporting on pregnancy rate among AGYW according to Moher et al.^41^ We limited studies to those published in English/French dating from 1 January 2007 to December 2017. We searched the following electronic databases: PubMed/MEDLINE, African Index Medicus and other African Journal online, EMBASE, Web of Science, Google Scholar, and Cochrane, using the following keywords: “adolescent pregnancy”, “youth pregnancy”, and “young women pregnancy” “teen pregnancy”. The PubMed advanced mesh search features used for example was: ((adolescent) OR adolescent pregnancy [MeSH]) AND ((pregnancy) OR (pregnancy adolescents) OR youth [MeSH] OR youth pregnancy [MeSH]) AND (((teen) OR teen pregnancy[MeSH] ) OR ((young women ) OR young women pregnancy A[MeSH])). Similarly, we searched using the following French key words: “grossesse et adolescents”, “grossesse et jeunes’’, “grossesse et jeunes femmes”. For example the Pubmed search English strategy used was “(“Young”[Journal] OR “young”[All Fields]) AND (“women”[MeSH Terms] OR “women”[All Fields]) AND (“pregnancy”[MeSH Terms] OR “pregnancy”[All ])” Fields]) OR “country specific” (tw))”. The search strategy was modified to include other online electronic databases such as BMC, Science Direct and PLoS One. Some of these studies were published as abstracts, conference posters and conference proceedings. Other data sources were national surveillance systems reporting on the rate of pregnancy among AGYW in some SADC countries. We embraced these sources as there were no specific pooled factor ratio describing the rate of pregnancy among AGYW between the ages of 10-24; 10-19 and 15-19 years in the 15 member states of the SADC Economic hub - a region with the highest unabated HIV reduction when compared to other region of the world.^18^

### Data collection and analysis

*Selection of studies*

Two independent review authors TM and SN extracted, screened the title and abstract results from the search online databases and applied pre-piloted checklist eligibility criteria to identify eligible studies. The articles were screened for duplicates, multiple publications, and other irrelevant studies under guidance of CY.

### Data extraction and management 

Using Microsoft Excel designed data extraction spreadsheet, TM and SN reviewed the extracted “ever pregnant” data among AGYW aged between 10-24 years on adolescent pregnancy”, “youth pregnancy”, and “young women pregnancy” “teen pregnancy”. The major data fields extracted were: country, age group and years, study design and recruitment methods, sample size, number pregnant, sample populationas shown inTable 1.^6,24,34,38,42-77^ The included age sub-groupings were: 10-24 years (25 studies),10-19 years (22 studies)and 15-19 years (12 studies). Only Somba et al^42^ had restricted data on subgrouping 19-23 years age (youth group). This was further reviewed by CY and AM for consistency. The rate of pregnancy from each study was extracted, together with the total number of participants enrolled, and the total number ever pregnant.

### Statistical methods

The AGYW pregnancy was defined as “ever been pregnant within the ages 10-24 years’’. We used the prevalence of pregnancy to measure the rate of pregnancy. We calculated prevalence for each included study by dividing the number of AGYW ever pregnant (n) by the total number of AGYW in the study sample and expressed it as percent.^63,78^

The sampling distribution for the prevalence statistic was assumed to have a normal distribution since the sample sizes were large enough to assume Central Limit Theorem.^63,78^ Using STATA 13.1 (StataCorp, Texas, USA), the prevalence of pregnancy from the different studies were pooled in a random effects meta-analysis since we anticipated heterogeneity owing to the studies in different countries and settings. We applied the I^2^ test statistic which estimates the percentage of variation that is due to heterogeneity rather than the chance occurrences, where values exceeding 50% indicate significant heterogeneity. We also applied the chi-square test to infer the extent of heterogeneity.^39^ In addition, the Egger’s regression test was used to estimate the study publication bias using metabias command.^79^ Results were displayed using forest plots. We investigated sources of heterogeneity through subgroup analysis with respect to the country from which the study was done and with respect to whether the study was from DHS.

## Results

### Study search results and characteristics of included studies

**Figure 1:**
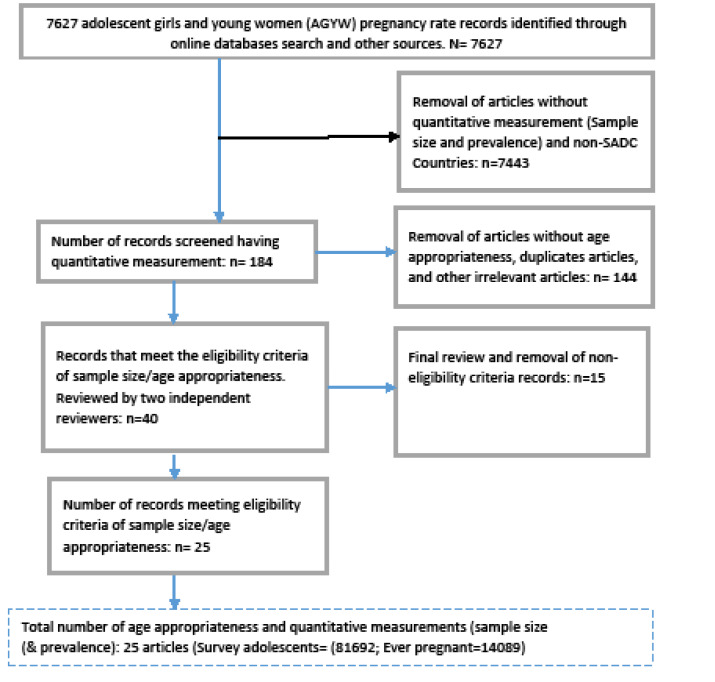
Identifying and screening of pregnancy rate articles among AGYW in SADC economic hub^41^

We identified a total of 7627 citations (7620 from electronic and 7 from other sources) reporting AGYW pregnancy from 2007 to 2017. We removed 7444 citations which were either not relevant or from non-SADC countries or had not reported quantitative data for the number of ever-pregnant AGYW. From the remaining 184 citations, 144 were out of the study age range (study age eligibility), duplicates and irrelevant articles. The remaining 40 articles were further subjected to review and 14 articles were non eligible due to study design (case control) or not reporting on study denominator (sample size) (Figure 1). Of the 7627 citations, only 25 studies were found eligible for age group 10-24 years of age,^60-62,66,68,69,71,74^ the majority from cross-sectional studies.^60-62,69^ Exploratory studies and DHS that reported only point prevalence without denominators or confidence intervals were also excluded. Qualitative studies were also excluded. Similarly, the non-experimental study from Zimbabwe had no denominator and was excluded.^75^ The following studies had no specific sample sizes: reporting pregnancy among adolescent in Botswana^76^ the Angola MIHS (2015-2016),^43^ and data from Zambia.^61^ The Mauritius teenage pregnancies of approximately 2000 cases per year arising from unsafe abortions^77^ was also excluded for the same reason. All the case control studies were ineligible and were excluded from the study.^59,61^ The female sex workers data from DRC was included because it met the inclusion criteria, having a sample size, prevalence and the population was 12-21 years of age^45^ (Table 1) despite the limitations.

Of the 15 countries in SADC economic hub: 13 countries had 25 eligible studies of pregnancy among AYGW aged 10-24 years freely available online-internet. The described 25 eligible studies were used to pool the national and regional pregnancy rate among AGYW. Of the 25 studies, 81 692 AGYW were enrolled, and of those 14 089 reported ever pregnant translating to a crude prevalence of 17.3% (95% CI, 12% to 18%).

### Pregnancy rate among adolescent girls and young women in SADC 

**Figure 2:**
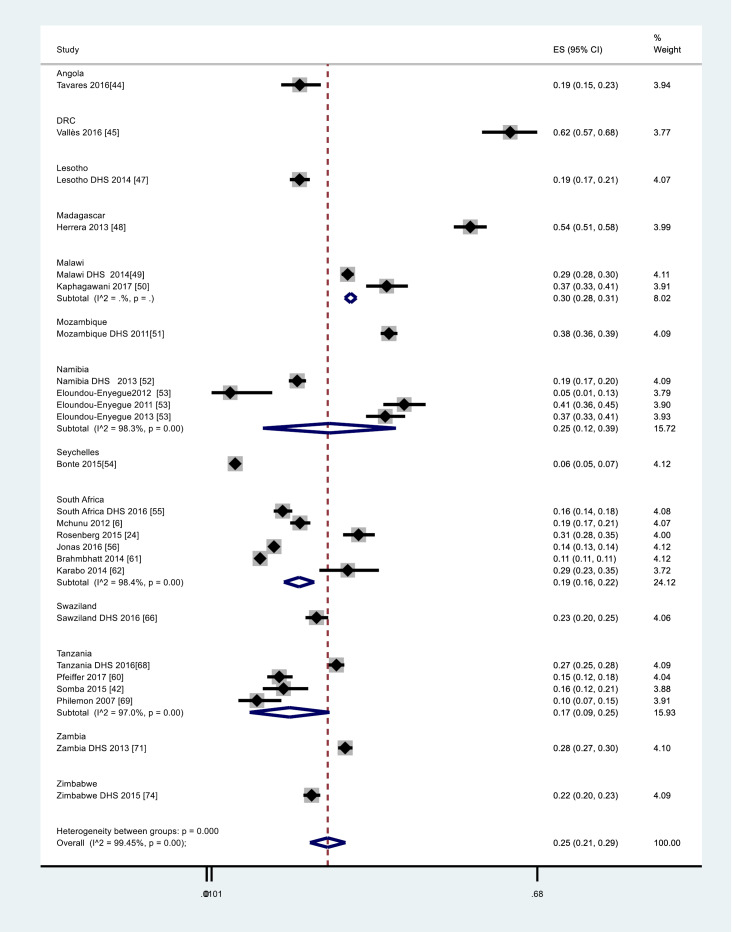
Forest plot for meta-analysis for rate of pregnancy among adolescent girls and young women (AGYW) aged 10-24 years from 2007-2017. Where the Y-axis described the weighted rate of pregnancy and the x-axis the log scale (0.0101 to 0.68). The proportion and the 95% CI of each study are indicated by the square and horizontal bar. The diamond estimated the pooled pregnancy proportion

A test of small study effects using metabias stata command found no significant small study effect (*P* = 0.900), indicating that there is no evidence of publication bias in the included studies of this systematic review. From the random effects meta-analysis, the overall regional weighted pregnancy rate (10-24 years)was estimated at 25% (95% CI: 21% to 28%). With regards to country specific weighted pregnancy rate, from highest to lowest, the overall meta-analysis results yielded pregnancy prevalence estimates as follows: DRC 62% (95% CI 57% to 68%), Madagascar 54% (95% CI: 51% to 58%), Mozambique 38% (95% CI: 36% to 39%), Malawi 30% (95% CI: 28% to 31%), Zambia 28% (95% CI 27% to 30%), Namibia 25% (95% CI: 12% to 39%), Swaziland 23% (95% CI: 20% to 25%), Zimbabwe 22% (95% CI: 20% to 23%), South Africa 19% (95% CI: 16% to 22%), Angola 19% (95% CI: 15% to 23%), Lesotho 19% (95% CI: 17% to 21%), Tanzania 17% (95% CI: 9% to 25%), Seychelles 6% (95% CI: 5% to 7%) as shown in Figure 2.^60-62,66,68,69,71,74^ The chi-square test was estimated at 1775.66, degrees of freedom (df) at 12 (*P* < 0.001) and the I-square statistic (I^2^) at 99.40% (*P* < 0.001) indicating significant heterogeneity between the country subgroups.

**Figure 3:**
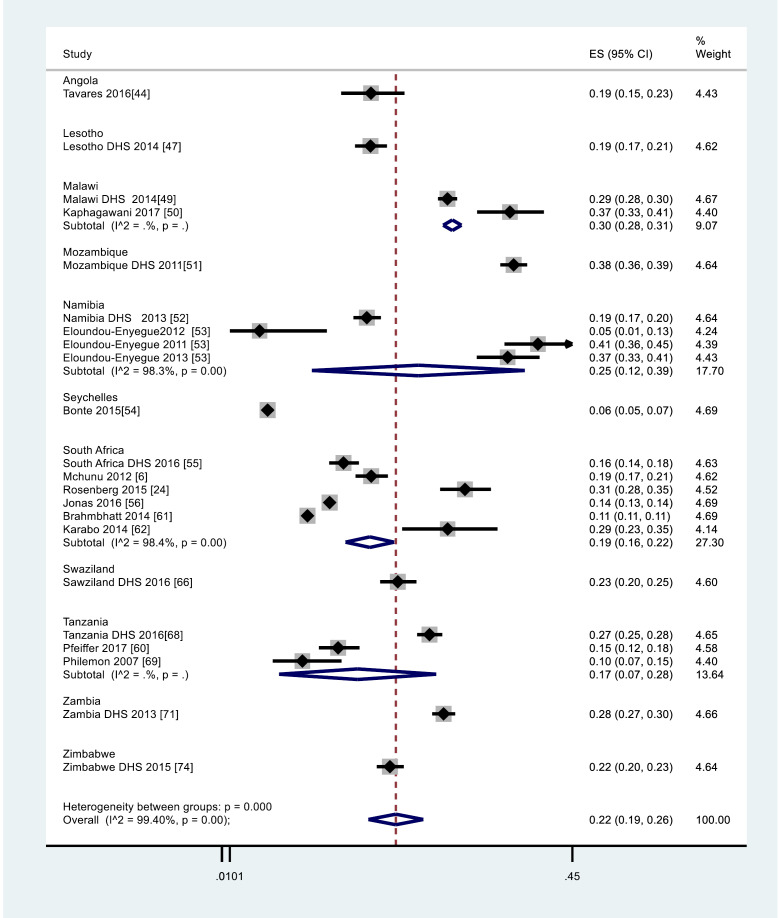
Forest plot for meta-analysis for rate of pregnancy among AGYW 10-19 years from 2007-2017. Where the Y-axis described the weighted rate of pregnancy and the x-axis the log scale (0.0101 to 0.45). The proportion and the 95% CI of each study are indicated by the square and horizontal bar. The diamond estimated the pooled pregnancy proportion

Those within the age range of 10-19 years (N = 80 287 vs n = 13 400) ever pregnant had a crude AGYW pregnancy rate of 16.7% ( 95% CI 16% to 17%) ) while the meta-analysis overall regional weighted ever pregnancy rate was estimated at 22% (95% CI: 19% to 26%) and nationally: Mozambique 38% (95% CI 36% to 39%), Malawi 30% (95% CI: 28% to 31%), Zambia 28% (95% CI 27% to 30%), Namibia 25% ( 95% CI: 12% to 39%), Swaziland 23% (95% CI: 20% to 25%), Zimbabwe 22% (95% CI: 20% to 23%), South Africa 19% (95% CI: 16% to 22%), Angola 19% (95% CI: 15% to 23%), Lesotho 19% (95% CI: 17% to 21%), Tanzania 17% (95%CI: 7% to 28%), Seychelles 6% (95% CI: 5% to 7%) as shown in Figure 3.^6,24,44,47-56,60-62,66,68,69,71,74^

**Figure 4:**
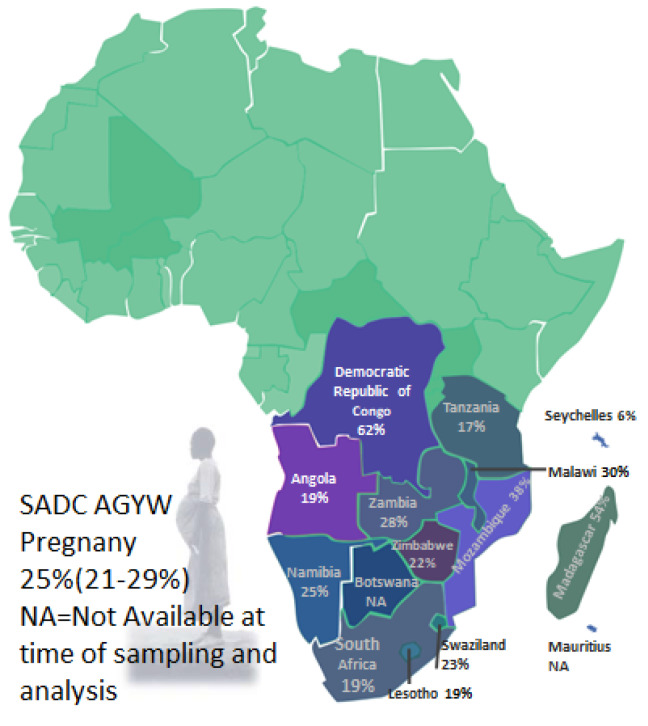
Map of Southern African Development Countries showing the distribution of pregnancy rate among AGYW aged 10-19 years.12 The data from Mauritius and Botswana will be updated whenever the data is available

Figure 4 describes the rate of pregnancy among AGYW aged 10-19 years scale across the SADC countries. The AGYW pregnancy rate was found to vary across the region as shown in Figure 4.^6,24,42,44-45,47-56,60-62,66,68,69,71,74^ The following countries (Botswana and Mauritius) were not included in Figure 4^76,77^ because of no age range data (Table 1).

**Figure 5:**
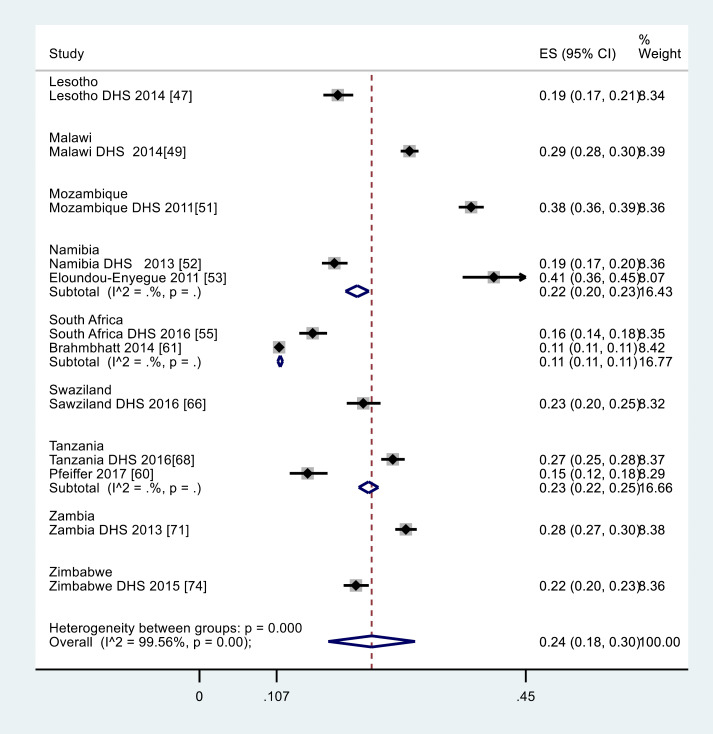
Forest plot for meta-analysis for rate of AGYW aged 15-19 years in SADC region from 2007-2017. The Y-axis described the weighted rate of pregnancy and the x-axis the log scale (0.125-0.45). The proportion and the 95% CI of each study are indicated by the square and horizontal bar. The diamond estimated the pooled pregnancy proportion

Figure 5 describes the weighted pregnancy rates of those 15-19 years. Only 9 of the 15 countries reported pregnancy rate of this age range resulting into an ever-pregnant number of 9916 from a sample size of 56 172 adolescents. The crude adolescent pregnancy rate was estimated at 17.6% (95% CI = 26% to 27%) while the meta-analysis overall regional weighted ever pregnancy rate was estimated at 24% (95% CI: 18% to 30%) and nationally: Mozambique 38% (95% CI 36% to 39%), Malawi 29% (95% CI: 28% to 30%), Zambia 28% (95% CI 27% to 30%), Swaziland 23% (95% CI: 20% to 25%), Tanzania 23% (95% CI: 22% to 25%), Zimbabwe 22% (95% CI: 20% to 23%), Namibia 22% ( 95% CI: 20% to 23%), Lesotho 19% (95% CI: 17% to 21%), South Africa 11% (95% CI: 11% to 11%) as indicated in Figure 5.^47,49,51-53,55,60,61,66,68,71,74^

## Discussion

High AGYW pregnancy and HIV/STIs new infections is of concern in the SADC region. This study was conducted to estimate the prevalence of pregnancy among AGYW aged 10-24, 10-19 and 15-19 years in the SADC region. The pregnancy evaluation was to enhance interventions that could enable the early linkage of AGYW to SRHR, contraceptive needs, and increase power to negotiate safer protected sex, proper use of contraceptives and reduction of HIV/STIs acquisition. Similarly, intensifying advocacy interventions that strengthen SRHR, family planning education and PMTCT for AGYW.^80,81^ For example, report shows that approximately one third of AGYW below 20 years of age of age attending ANC in South Africa are HIV positive.^34,82^ High HIV seroconversion during late pregnancy has been witnessed among women in the general population, enhancing vertical MTCT of HIV.^83^ In addition, a study conducted in 2017 in Harare, Zimbabwe showed that out of 1786 HIV positive women attending PMTCT, 1756 (98%) knew their HIV status and 179 (11%) were without documented viral load records at PMTCT.^83^ Also, mental health is one of the factors limiting AGYW attending PMTCT.^84^ According to a study by Nyamukoho et al,^84^ HIV positive AGYW attending PMTCT were 3.2 times more likely to suffer from depression, compared to older women.Therefore, detailed age appropriate epidemiological data is needed to provide an accurate estimate of pregnancy rate among AGYW which can be used to enhance interventions towards mental health interventions.

Our results showed a varied national pregnancy rate among AGYW in SADC countries. This result was similar to other low-income countries such as Latin America and the Caribbean where adolescent fertility remains high.^37^ The same report^37^ documents measures to reduce AGYW pregnancy, such as joint regional commitment task force, sub regional and national action plans targeted age appropriate disaggregation by engaging and empowering youth to contribute and drive the design, implementation, and monitoring of interventions. Our results show the pooled pregnancy rate for AGYW aged 15-19 years at 24% and national variation from 11% in South Africa to 38% in Zambia. The national variation for Latin America and the Caribbean was between 11.6% in Uruguay to 30.7% in Panama.^37^ To reduce AGYW pregnancy by 20%, UNFPA^85^ has instigated the leveraging of proven and effective interventions such as access to effective contraceptives, comprehensive sexual education (CSE), age-appropriate counselling, access to available, equitable and acceptable information, building and creating gender equality, adolescent SRHR education at targeted settings. In addition, reports by Yakubu et al^13^ and UNICEF^80^ also highlight awareness and knowledge regarding sexual education, health and family planning, and parenting skill development that include, contraception methods, and SRH misconceptions. Although, contraception misconceptions are likely to differ in some settings due to differences between modern and traditional attitudes towards contraceptive methods.^86-89^

In some settings, the majority of AGYW do not make use of contraception methods.^80^ For example, in settings such as India, IIPS^90^ conducted a household survey in 2008 among AGYW aged 15-24 years and found that more than half of the AGYW population had never received any formal education about sex or family planning. In the same study 77% of the girls had no formal contraception education.^90^ Sexual and gender-based violence (SGBV) and gender inequalities are some of the root causes of the high pregnancy rate in the region.^34^ Based on these limitations, the SADC Maputo Protocol of 2016 was held to harmonize national abortion and pregnancy laws and policies in Southern and Eastern Africa.^91^ The meeting proceedings advocated for more research and interventions by expanding women comprehensive sexual and reproductive education and health services.

With respect to AGYW accessing health care for contraceptives and SRHR information, many SADC countries have rolled-out adolescent friendly youth services including SRHR information.^75,92^ However, AGYW are still confronted with negative and stigmatizing attitudes of health care providers when seeking health care.^8,13,93^ Due to these challenges and according to report by Panday,^94^ AGYW delay access to health care services during pregnancy^94,95^ resulting in five times SRH consequences among adolescents girls less than 15 years of age when compared to older women^96^

The pooled estimate of pregnancy rate among AGYW in South Africa and Zimbabwe was estimated at 16% and 22%. This was slightly less than the 30% reported by Flanagan et al^97^ and Willan et al^98^ among AGYW who had ‘ever been pregnant’. This number decreased but still high.^97,98^ According to reports by Lillian et al,^99^ high AGYW pregnancy in Namibia is influenced by educational background, socio-economic status, and cultural beliefs. In response to these challenges the Namibian government has geared intervention programs and policy towards youth CSE regardless of the socio-economic and/or cultural contexts and status. In Mozambique, Decree 39/GM/2003 has been initiated where pregnant schoolgirls are initiated on extra mural studies in order to complete their education.^100^

This review showed that DRC has the highest pregnancy rate, estimated at 62%: this correlates with the country’s high birth rate. DRC is the highest populated country in SADC region and the 3rd populated country in sub Saharan Africa with an estimated population of over 80 million people and a fertility rate of 6.1% per woman.^101^

Swaziland has the highest rate of HIV among women of 15-49 (35.1%) and adolescents (16.7%) globally^102^ and the pregnancy rate was estimated at 22% among adolescents aged 10-19 years.

AGYW who desire to be pregnant are less likely to practice safer sex. This was highlighted by findings from the Mozambique DHS showing AGYW who want to get pregnant were less likely to use condoms and other prevention methods with non-marital partners than those who want to delay childbearing.^103^ Furthermore, according to reports by Neal et al,^104^ approximately 2.5 million AGYW give birth before the age of 16 each year in low resource settings and around 50%–65% of them before the age of 15 especially in Chad, Guinea, Mali, and Mozambique. Due to the increased risks of engaging in unsafe sexual intercourse among unmarried AGYW: identifying and scaling up evidence-based programs for sexually active AGYW is critical. Positive cost-effective ways of addressing and reshaping AGYW CSE, access to SRHR, and use of pre exposure prophylaxis (PrEP). Pregnancy itself does not increase the risk of HIV/STI acquisition, however unprotected sex, a singular incident of pregnancy acquisition plays a significant role of HIV/STI transmission. It should be noted that AGYW pregnancy has been a neglected research area despite the risks associated with HIV/AIDS, sexual abuse, infant and maternal mortality, school drop-out, and loss of self-esteem. The study suggests the strengthening of SRHR and CSE programs among AGYW as part of the care package during clinical and ANC visits.

According to a report by Higgins et al,^40^ there is always potential biases in study reviews, including agreements and disagreements, applicability of evidence and quality of the evidence.^40,105^ Only English and French were used as the language of study search. There was no relevant data from Botswana and Mauritius and the sex worker data from DRC skewed the outcome of 10-24 years of age. The studies were not also aggregated as urban and rural areas limiting setting generalizability. Due to publication bias, findings with favourable outcomes may not have been available especially those of Population Council, Guttmacher and WHO. Despite these limitations, the search generated real time generalizable effect size estimates. The majority of the cross-sectional studies included had adjusted key variables. In addition, adolescent pregnancy data between those aged 15-19 years were mostly from DHS (Figure 5), increasing the power and generalization of the findings. The regression analysis showed that the combined constant was 0.900 and the *P* value at 0.900 indicating no publication bias for those 10-24 years of age.

## Conclusion and Recommendation

The study revealed a high pregnancy rate among AGYW in the SADC region. The high pooled country specific and regional rate among AGYW has highlighted the need for informed SRHR policies and programmes to be tailored towards AGYW. This includes policy changes that will improve the collaboration between adolescents, health care providers, parents, and teachers to address adolescent sexuality education. In addition, increased research is needed to explore innovative ways to expand and improve sexuality communication and sexuality risk reduction strategies that expose adolescents to unsafe sexual practices, unwanted pregnancies, HIV/STIs and improve health seeking behaviour. The reduced focus on the pregnancy rate among AGYW affects the social, political and economic development of the region.

## Acknowledgments

We acknowledge the postgraduate and intern students that assisted in the extraction of the data especially Tetelo Maakamedi (TM) our intern student who helped in the the data extraction and Dr. Alfred Musekiwa (AM) who opted and carefully reviewed the meta-statistics. The study had no funding support.

## Competing interests

None declared.

## Ethical approval

Since all data used in this systematic review have already been published or available in the public domain, there was no ethical clearance required for this review.

## Authors’ contributions

CSY conceptualized, designed, reviewed the data, analysed and wrote the initial draft of the article. TM, SN extracted and reviewed the eligibility criteria. Differences or disagreements between the reviewing authors were resolved through a discussion meeting by a third party (CSY). CSY conducted and performed the meta-analysis statistics which was further reviewed by AM and advised on the general methods and structure. CSY, SN, NN, TM, AK and SM gave further interpretation and critical appraisal.

## Disclaimer

The findings, interpretations, and conclusions expressed in this publication do not necessarily reflect the views of any regional or national governments but represent those of the researchers.

**Table 1:** The age range, study designs, burden of pregnancy among adolescent girls and young women (AGYW) across Southern African Development Community (SADC) economic hub: A systematic review

**Country**	**Age group (y)**	**Study design**	**Sample size**	**Number pregnant**	**Included (Yes) or Excluded (No)**	**Reference**
Angola	15-19	Multiple Indicator Health Survey (MIHS)	NA	NA	No	^43^
Angola	10-19	Cross-sectional study	381	73	Yes	^44^
DRC	12-21	Cross-sectional study	293	183	Yes	^45^
DRC	15-19	DHS	NA	Not well defined	No	^46^
Lesotho	15-19	DHS	1440	275	Yes	^47^
Madagascar	14-23	Cross-sectional study	859	466	Yes	^48^
Malawi	15-19	DHS	5263	1526	Yes	^49^
Malawi	10-19	Cross-sectional study	505	187	Yes	^50^
Mozambique	15-19	DHS	3061	1148	Yes	^51^
Namibia	15-19	DHS	1906	355	Yes	^52^
Namibia	Adolescents	Cross-sectional study	220	43	No	^38^
Namibia	10-14	Cross-sectional study	62	3	Yes	^53^
Namibia	15-19	Cross-sectional study	507	206	Yes	^53^
Namibia	10-19	Cross-sectional study	569	209	Yes	^53^
Seychelles	12-19	Cross-sectional study	4497	264	Yes	^54^
South Africa	15-19	DHS	1427	223	Yes	^55^
South Africa	12-19	Cross-sectional study	1417	272	Yes	^6^
South Africa	Adolescents	Cross-sectional study	800	250	Yes	^24^
South Africa	12-18	Cross-sectional study	15457	2140	Yes	^56^
South Africa	Adolescent school girls	Cross-sectional study(not within inclusion year)	1025	214	No	^57^
South Africa	15-19	Cross-sectional study (not within inclusion year)	283	67	No	^58^
South Africa	<19	A retrospective cohort study	Not clearly defined	1236	No	^59^
South Africa	< 19	Case-control study	353	191	No	^60^
South Africa	11-19	Cross-sectional study	31816	3500	Yes	^61^
South Africa	11-19	Cross-sectional study	224	65	Yes	^62^
South Africa	Adolescents	Cross-sectional study	582	221	Yes	^63^
South Africa	15-19	Cross-sectional study	283	67	Yes	^34^
South Africa	> 19 years older	NA	7838 all deliveries	1236 from teenage girls	No	^64^
South Africa		Case control	544	199 cases	No	^65^
Swaziland	15-19	DHS	1274	288	Yes	^66^
Swaziland	15-19	Exploratory	All 33 pregnant	33	No	^67^
Tanzania	15-19	DHS	2904	775	Yes	^68^
Tanzania	15-19	Cross-sectional study	750	112	Yes	^60^
Tanzania	19-23	Cross-sectional study	253	40	Yes	^42^
Tanzania	10-19	Cross-sectional study	203	21	Yes	^69^
Tanzania	No really specified	Case control	Not clearly defined	190	No	^59^
Tanzania	<15-19	Unmatched case control	354	190	No	^70^
Zambia	15-19	DHS	3625	1033	Yes	^71^
Zambia	13-19	Cases control	400	200 cases	No	^72^
Zimbabwe	14-19	Non -experimental descriptive	All 80 pregnant	80	No	^73^
Zimbabwe	15-19	DHS	2199	475	Yes	^74^
Zimbabwe	14-19	-	80		No	^75^
Botswana	School age	(Botswana Government, 2007	Not clearly defined	324	No	^76^
Mauritius	15-19	Fact sheet (2006-2011)	Not clearly defined	NA	No	^77^

## References

[1] Odenigbo C, Cooper S, Comeau N, Dorel L, Forster K, Kinman A, et al. MonWHO Theme Guide 2017: Sexual Health. Geneva: Montréal World Health Organization Simulation; 2017.

[2] Haffejee F, O’Connor L, Govender N, Reddy P, Sibiya MN, Ghuman S (2018). Factors associated with unintended pregnancy among women attending a public health facility in KwaZulu-Natal, South Africa. South African Family Practice.

[3] Harrison A, Smit J, Hoffman S, Nzama T, Leu CS, Mantell J (2012). Gender, peer and partner influences on adolescent HIV risk in rural South Africa. Sex Health.

[4] Hubacher D, Mavranezouli I, McGinn E (2008). Unintended pregnancy in sub-Saharan Africa: magnitude of the problem and potential role of contraceptive implants to alleviate it. Contraception.

[5] Lundeen T (2016). Intrapartum and postpartum transfers to a tertiary care hospital from out-of-hospital birth settings: a retrospective case series. J Midwifery Womens Health.

[6] Mchunu G, Peltzer K, Tutshana B, Seutlwadi L (2012). Adolescent pregnancy and associated factors in South African youth. Afr Health Sci.

[7] Nance N, Ralph L, Padian N, Cowan F, Buzdugan R, Mushavi A (2018). Unintended pregnancy and subsequent postpartum long-acting reversible contraceptive use in Zimbabwe. BMC Womens Health.

[8] Pizzol D, Di Gennaro F, Boscardin C, Putoto G, Cuppini E, Pita G (2018). Teenage pregnancies in Mozambique: the experience of “Servicios Amigos dos Adolescentes” clinics in Beira. Afr J AIDS Res.

[9] Remez L, Woog V, Mhloyi M. Sexual and Reproductive Health Needs of Adolescents in Zimbabwe. Available from: https://www.guttmacher.org/sites/default/files/report_pdf/ib-zimbabwe_0.pdf. Accessed April 14, 2019. 26159001

[10] Santhya KG, Jejeebhoy SJ (2015). Sexual and reproductive health and rights of adolescent girls: evidence from low- and middle-income countries. Glob Public Health.

[11] Schuyler AC, Masvawure TB, Smit JA, Beksinska M, Mabude Z, Ngoloyi C (2016). Building young women’s knowledge and skills in female condom use: lessons learned from a South African intervention. Health Educ Res.

[12] Yah CS, Tambo E, Adeagbo O, Magida A (2017). HIV and sexually transmitted co-infections among sex workers in the Southern African economic region. Ann Trop Med Public Health.

[13] Yakubu I, Salisu WJ (2018). Determinants of adolescent pregnancy in sub-Saharan Africa: a systematic review. Reprod Health.

[14] Honig AS (2012). Teen pregnancy. Int J Adolesc Youth.

[15] McCleary-Sills J, Douglas Z, Rwehumbiza A, Hamisi A, Mabala R (2013). Gendered norms, sexual exploitation and adolescent pregnancy in rural Tanzania. Reprod Health Matters.

[16] Conde-Agudelo A, Belizán JM, Lammers C (2005). Maternal-perinatal morbidity and mortality associated with adolescent pregnancy in Latin America: cross-sectional study. Am J Obstet Gynecol.

[17] Gilbert W, Jandial D, Field N, Bigelow P, Danielsen B (2004). Birth outcomes in teenage pregnancies. J Matern Fetal Neonatal Med.

[18] Grønvik T, Fossgard Sandøy I (2018). Complications associated with adolescent childbearing in sub-Saharan Africa: a systematic literature review and meta-analysis. PLoS One.

[19] Klein JD (2005). Adolescent pregnancy: current trends and issues. Pediatrics.

[20] United Nations Population Fund (UNFPA). Status Report on Adolescents and Young People in Sub-Saharan Africa: Opportunities and Challenges. UNFPA-Regional office Johannesburg; 2012. Available from: https://esaro.unfpa.org/en/publications/status-report-adolescents-and-young-people-sub-saharan-africa. Accessed November 2017.

[21] World Health Organization (WHO). Adolescent Pregnancy [‎Electronic Resource]‎: Unmet Needs and Undone Deeds: A Review of the Literature and Programmes. Geneva: WHO; 2007.

[22] Southern African Development Community (SADC). SADC HIV and AIDS Strategic Framework 2010-2015. SADC; 2016. Available from: https://www.sadc.int/files/4213/5435/8109/SADCHIVandAIDSStrategyFramework2010-2015.pdf. Accessed November 2017.

[23] Mason-Jones AJ, Sinclair D, Mathews C, Kagee A, Hillman A, Lombard C (2016). School-based interventions for preventing HIV, sexually transmitted infections, and pregnancy in adolescents. Cochrane Database Syst Rev.

[24] Rosenberg M, Pettifor A, Miller WC, Thirumurthy H, Emch M, Afolabi SA (2015). Relationship between school dropout and teen pregnancy among rural South African young women. Int J Epidemiol.

[25] Chigona A, Chetty R. Girls’ education in South Africa: special consideration to teen mothers as learners. J Educ Int Dev 2007; 3(1); 17.

[26] Fleischman J, Peck K. Addressing HIV Risk in Adolescent Girls and Young Women. Center for Strategic and International Studies (CSIS); 2015. Available from: https://www.csis.org/analysis/addressing-hiv-risk-adolescent-girls-and-young-women. Accessed May 2017.

[27] Govender C. Experiences of Teenage Mothers in the Informal Settlements: An Analysis of Young Females’ Reproductive Health Challenges, A Case Study of Siyanda Informal Settlement [dissertation]. Durban: University of KwaZulu-Natal; 2012.

[28] Kharsany AB, Karim QA (2016). HIV infection and AIDS in sub-Saharan Africa: current status, challenges and opportunities. Open AIDS J.

[29] Magowe MKM, Seloilwe E, Dithole K, St Lawrence J (2017). Perceptions of key participants about Botswana adolescents’ risks of unplanned pregnancy, sexually transmitted diseases, and HIV: qualitative findings. Jpn J Nurs Sci.

[30] Manzini N (2001). Sexual initiation and childbearing among adolescent girls in KwaZulu Natal, South Africa. Reprod Health Matters.

[31] McGrath N, Nyirenda M, Hosegood V, Newell ML (2009). Age at first sex in rural South Africa. Sex Transm Infect.

[32] UNAIDS. Putting HIV Prevention Among Adolescent Girls and Young Women on the Fast-Track and Engaging Men and Boys. UNAIDS; 2016. Available from: https://www.unaids.org/en/resources/presscentre/featurestories/2015/february/20150211_africanleaders . Accessed March 25, 2017.

[33] UNAIDS. African Leaders Reaffirm Commitment to the AIDS Response and Women’s Empowerment. Eastern and Southern Africa; 2015. Available from: https://www.unaids.org/en/resources/presscentre/featurestories/2015/february/20150211_africanleaders .Accessed March 25, 2017.

[34] Dellar RC, Dlamini S, Karim QA (2015). Adolescent girls and young women: key populations for HIV epidemic control. J Int AIDS Soc.

[35] Abdool Karim Q, Kharsany AB, Leask K, Ntombela F, Humphries H, Frohlich JA (2014). Prevalence of HIV, HSV-2 and pregnancy among high school students in rural KwaZulu-Natal, South Africa: a bio-behavioural cross-sectional survey. Sex Transm Infect.

[36] Shisana O, Rehle T, Simbayi LC, Zuma K, Jooste S, Zungu N, et al. South African National HIV Prevalence, Incidence and Behaviour Survey, 2012. Cape Town, South Africa: HSRC Press; 2014. 10.2989/16085906.2016.1153491 27002359

[37] Caffe S, Plesons M, Camacho AV, Brumana L, Abdool SN, Huaynoca S (2017). Looking back and moving forward: can we accelerate progress on adolescent pregnancy in the Americas?. Reprod Health.

[38] Trussell J, Henry N, Hassan F, Prezioso A, Law A, Filonenko A (2013). Burden of unintended pregnancy in the United States: potential savings with increased use of long-acting reversible contraception. Contraception.

[39] Higgins JPT, Altman DG, Sterne JAC. Chapter 8: Assessing risk of bias in included studies. In: Higgins JPT, Green S, eds. Cochrane Handbook for Systematic Reviews of Interventions. Chichester, UK: John Wiley & Sons; 2008.

[40] Higgins JP, Altman DG, Gøtzsche PC, Jüni P, Moher D, Oxman AD (2011). The Cochrane Collaboration’s tool for assessing risk of bias in randomised trials. BMJ.

[41] Moher D, Liberati A, Tetzlaff J, Altman DG (2009). Preferred reporting items for systematic reviews and meta-analyses: the PRISMA statement. PLoS Med.

[42] Somba MJ, Mbonile M, Obure J, Mahande MJ (2014). Sexual behaviour, contraceptive knowledge and use among female undergraduates’ students of Muhimbili and Dar es Salaam Universities, Tanzania: a cross-sectional study. BMC Womens Health.

[43] Multiple Indicator Health Survey (MIHS), Instituto Nacional de Estatística (INE) MdSM, Ministério da Planeamento e do Desenvolvimento Territorial (MPDT), ICF. Key Findings of the 2015-16 Angola IIMS. Rockville, Maryland, USA: INE, MINSA, MPDT and ICF; 2017. Available from: https://dhsprogram.com/pubs/pdf/SR238/SR238.pdf. Accessed July 14, 2018.

[44] Dos Prazeres Tavares H, Tavares SB, Capingana DP, da Gama SG, da Silva LG (2016). Obstetric, sociodemographic, and psychosocial problems of postpartum adolescents of Huambo, Angola. Clin Med Insights Womens Health.

[45] Vallès X, Lusala PL, Devalière H, Metsia-Thiam MM, Aguilar D, Cheyron AL (2017). Network analysis of knowledge and practices regarding sexual and reproductive health: a study among adolescent street girls in Kinshasa (DRC). Eur J Contracept Reprod Health Care.

[46] DRC Demographic Health Survey (DHS). Ministère du Plan et Suivi de la Mise en œuvre de la Révolution de la Modernité - MPSMRM/Congo MdlSP-MCaII. Enquête Démographique et de Santé en République Démocratique du Congo 2013-2014. Rockville, Maryland, USA: MPSMRM, MSP, ICF International; 2014.

[47] Ministry of Health and Social Welfare, ICF Macro. Lesotho Demographic and Health Survey 2009. Maseru, Lesotho: Ministry of Health and Social Welfare, ICF Macro; 2010. Available from: https://dhsprogram.com/pubs/pdf/FR241/FR241.pdf. Accessed February 28, 2018.

[48] Herrera C, Sahn DE, Villa KM. Teen Fertility and Labor Market Segmentation in Madagascar. Abidjan, Côte d’Ivoire: African Development Bank; 2017.

[49] National Statistics Office, The DHS Program ICF. Malawi: Demographic and Health Survey, 2015-16. 2017. Zomba, Malawi: National Statistics Office, The DHS Program ICF; 2017.

[50] Kaphagawani NC, Kalipeni E (2017). Sociocultural factors contributing to teenage pregnancy in Zomba district, Malawi. Glob Public Health.

[51] Mozambique Demographic Health Survey (DHS). Ministério da Saúde- MISAU INdE-I, and ICF. Inquérito de Indicadores de Imunização, Malária e HIV/SIDA em Moçambique - IMASIDA, 2015. Maputo/Moçambique: MISAU/Moçambique, INE, ICF; 2016. Available from: https://dhsprogram.com/publications/publication-ais12-ais-final-reports.cfm. Accessed February 28, 2018.

[52] Ministry of Health and Social Services, Namibia Statistics Agency. Namibia: Demographic and Health Survey, 2013. Windhoek, Namibia: Ministry of Health and Social Services, Namibia Statistics Agency; 2014. Available from: https://cospaceoxford.org. Accessed February 28, 2018.

[53] Eloundou-Enyegue P, Magazi S. Teenage pregnancy in Kavango region: Contributing factors and program: Recommendations. A Policy Study conducted for the United States Agency for International Development. 2011. Available from: http://pdf.usaid.gov/pdf_docs/pnady831.pdf. Accessed February 28, 2018.

[54] World Bank Group, World Development Indicators; Adolescent fertility rate (births per 1,000 women ages 15-19). Available from: https://data.worldbank.org/indicator/SP.ADO.TFRT?locations=SC. Accessed 15 December 2017.

[55] South Africa Demographic and Health Survey (DHS) 2016. National Department of Health - NDoH SSA-SS, South African Medical Research Council - SAMRC, and ICF. Pretoria, South Africa: NDoH, Stats SA, SAMRC, ICF; 2019. Available from: https://dhsprogram.com/pubs/pdf/FR337/FR337.pdf. Accessed February 28, 2018.

[56] Jonas K, Crutzen R, van den Borne B, Sewpaul R, Reddy P (2016). Teenage pregnancy rates and associations with other health risk behaviours: a three-wave cross-sectional study among South African school-going adolescents. Reprod Health.

[57] Buga GA, Amoko DH, Ncayiyana DJ (1996). Adolescent sexual behaviour, knowledge and attitudes to sexuality among school girls in Transkei, South Africa. East Afr Med J.

[58] Malema RN. Risk Factors Associated with Teenage Pregnancy at Ga-Dikgale Villages in the Northern Province of South Africa [dissertation]. Pretoria: University of Pretoria; 2010.

[59] Lukolo LN. Adolescent Sexual Health in A Selected Region of Namibia [dissertation]. Stellenbosch: Stellenbosch University; 2001.

[60] Pfeiffer C, Ahorlu CK, Alba S, Obrist B (2017). Understanding resilience of female adolescents towards teenage pregnancy: a cross-sectional survey in Dar es Salaam, Tanzania. Reprod Health.

[61] Brahmbhatt H, Kågesten A, Emerson M, Decker MR, Olumide AO, Ojengbede O (2014). Prevalence and determinants of adolescent pregnancy in urban disadvantaged settings across five cities. J Adolesc Health.

[62] Karabo M, Ayiga N (2014). Rates and predictors of school pregnancy among black women in the North West province, South Africa. African Population Studies.

[63] Boyd R, Jeong K, Tomé WA (2019). Determining efficient helical IMRT modulation factor from the MLC leaf-open time distribution on precision treatment planning system. J Appl Clin Med Phys.

[64] Hoque M, Hoque S (2010). A comparison of obstetrics and perinatal outcomes of teenagers and older women: experiences from rural South Africa. Afr J Prim Health Care Fam Med.

[65] Vundule C, Maforah F, Jewkes R, Jordaan E (2001). Risk factors for teenage pregnancy among sexually active black adolescents in Cape Town. A case control study. S Afr Med J.

[66] Central Statistical Office, Macro International. Swaziland: Demographic and Health Survey, 2006-2007. Mbabane, Swaziland: Central Statistical Office, Macro International; 2008.

[67] Mngadi PT, Zwane IT, Ransjö-Arvidson AB, Ahlberg BM (2002). Quality of maternity care for adolescent mothers in Mbabane, Swaziland. Int Nurs Rev.

[68] Tanzania Demographic and Health Survey and Malaria Indicator Survey (TDHS-MIS) 2015-2016. Ministry of Health CD, Gender, Elderly and Children (MoHCDGEC) Tanzania Mainland, Ministry of Health (MoH) Zanzibar, National Bureau of Statistics (NBS), Office of the Chief Government Statistician (OCGS), and ICF. 2016. Dar es Salaam, Tanzania, and Rockville, Maryland, USA: MoHCDGEC, MoH, NBS, OCGS, and ICF. Available from: https://dhsprogram.com/pubs/pdf/FR321/FR321.pdf. Accessed 15 March 2017.

[69] Philemon MN. Factors Contributing to High Adolescent Pregnancy Rate in Kinondoni Municipality. Dar es Salaam, Tanzania: University of South Africa; 2007.

[70] Lyamuya RE. Obstetric Outcome Among Adolescent Primigravidae Delivering at Muhimbili National Hospital Dar es Salaam, Tanzania [dissertation]. Dar es Salaam: Muhimbili University; 2002.

[71] Zambia Demographic and Health Survey (DHS) 2013-14. Central Statistical Office/Zambia MoHZ, and ICF International. 2014. Rockville, Maryland, USA: Central Statistical Office/Zambia, Ministry of Health/Zambia, and ICF International. Available from: https://dhsprogram.com/publications/publication-fr304-dhs-final-reports.cfm. Accessed 14 May 2018.

[72] Kumwenda A. Determinants of Teenage Pregnancy in Lusaka District [dissertation]. Lusaka, Zambia: University of Zambia; 2010.

[73] Chaibva CN, Roos JH, Ehlers VJ (2009). Adolescent mothers’ non-utilisation of antenatal care services in Bulawayo, Zimbabwe. Curationis.

[74] International ZNSAaI. Zimbabwe Demographic and Health Survey (DHS) 2015: Final Report. 2016. Rockville, Maryland, USA: Zimbabwe National Statistics Agency (ZIMSTAT) and ICF International. Available from: https://dhsprogram.com/publications/publication-fr322-dhs-final-reports.cfm. Accessed 18 January 2017.

[75] Bukenya JN, Mulogo E, Kibira SPS, Muhumuza C, Atuyambe LM. Health facilities’ readiness to provide friendly reproductive health services to young people aged 10-24 years in Wakiso district, Uganda. Glob J Reprod Med 2017;2(3). 10.19080/gjorm.2017.02.555588 PMC6106767 30148262

[76] Molosiwa S, Moswela B (2012). Girl-pupil dropout in secondary schools in Botswana: influencing factors, prevalence and consequences. Int J Bus Soc Sci.

[77] HEARD. Country Factsheet: Mauritius. Durban: University of KwaZulu-Natal; 2015. Available from: https://www.heard.org.za/. Accessed February 28, 2018.

[78] Ball F, Britton T, Leung KY, Sirl D (2019). A stochastic SIR network epidemic model with preventive dropping of edges. J Math Biol.

[79] Egger M, Davey Smith G, Schneider M, Minder C (1997). Bias in meta-analysis detected by a simple, graphical test. BMJ.

[80] UNICEF. Early Marriage: A Harmful Traditional Practice. UNICEF; 2005. Available from: https://www.unicef.org/publications/index_26024.html. Accessed February 28, 2018.

[81] Yah CS, Tambo E (2019). Why is mother to child transmission (MTCT) of HIV a continual threat to new-borns in sub-Saharan Africa (SSA). J Infect Public Health.

[82] Manyahi J, Jullu BS, Abuya MI, Juma J, Kilama B, Sambu V (2017). Decline in the prevalence HIV among pregnant women attending antenatal clinics in Tanzania, 2001-2011. Tanzan J Health Res.

[83] Komtenza B, Satyanarayana S, Takarinda KC, Mukungunugwa SH, Mugurungi O, Chonzi P (2019). Identifying high or low risk of mother to child transmission of HIV: How Harare city, Zimbabwe is doing?. PLoS One.

[84] Nyamukoho E, Mangezi W, Marimbe B, Verhey R, Chibanda D (2019). Depression among HIV positive pregnant women in Zimbabwe: a primary health care based cross-sectional study. BMC Pregnancy Childbirth.

[85] UNFPA. Integrated Strategic framework for the reduction of adolescent Pregnancy in the Caribbean (2014-2019). 2019. UNFPA Sub-regional office for the Caribbean’s. Available from: https://caribbean.unfpa.org/en/news/adolescent-pregnancy-not-just-health-issue-unfpa-0. Accessed 12 April 2017.

[86] Abdul-Rahman L, Marrone G, Johansson A (2011). Trends in contraceptive use among female adolescents in Ghana. Afr J Reprod Health.

[87] Kabagenyi A, Reid A, Ntozi J, Atuyambe L (2016). Socio-cultural inhibitors to use of modern contraceptive techniques in rural Uganda: a qualitative study. Pan Afr Med J.

[88] May JF, Mukamanzi M, Vekemans M (1990). Family planning in Rwanda: status and prospects. Stud Fam Plann.

[89] Nguyen MC, Wodon Q (2015). Global and regional trends in child marriage. Rev Faith Int Aff.

[90] International Institute for Population Sciences (IIPS). District Level Household and Facility Survey (DLHS-3), 2007-08. Mumbai, India; IIPS; 2010.

[91] Thompson J, Undie CC, Amin A, Johnson BR, Khosla R, Ouedraogo L (2018). Harmonizing national abortion and pregnancy prevention laws and policies for sexual violence survivors with the Maputo Protocol: proceedings of a 2016 regional technical meeting in sub-Saharan Africa. BMC Proc.

[92] Mendelsohn AS, Gill K, Marcus R, Robbertze D, van de Venter C, Mendel E (2018). Sexual reproductive healthcare utilisation and HIV testing in an integrated adolescent youth centre clinic in Cape Town, South Africa. South Afr J HIV Med.

[93] Mulaudzi M, Dlamini BN, Coetzee J, Sikkema K, Gray G, Dietrich JJ (2018). Perceptions of counsellors and youth-serving professionals about sexual and reproductive health services for adolescents in Soweto, South Africa. Reprod Health.

[94] Panday S, Makiwane M, Ranchod C, Letsoala T. Teenage pregnancy in South Africa: With A Specific Focus on School-Going Learners. South Africa: Human Sciences Research Council (HSRC); 2009.

[95] Nove A, Matthews Z, Neal S, Camacho AV (2014). Maternal mortality in adolescents compared with women of other ages: evidence from 144 countries. Lancet Glob Health.

[96] Rowbottom S. Giving Girls Today & Tomorrow: Breaking the Cycle of Adolescent Pregnancy. New York: United Nations Population Fund; 2007.

[97] Flanagan A, Lince N, Durao de Menezes I. Teen Pregnancy in South Africa: A Literature Review Examining Contributing Factors and Unique Interventions. Ibis Reproductive Health; 2013. Available from: http://www.mmoho.co.za/wp-content/uploads/2016/02/Literature-Review_Teenage-Pregnancy-South-Africa_Ibis.pdf. Accessed June 2019.

[98] Willan S. A Review of Teenage Pregnancy in South Africa – Experiences of Schooling, And Knowledge and Access to Sexual and Reproductive Health Services. UNESCO; 2013. Available from: https://www.hst.org.za/publications/NonHST%20Publications/Teenage%20Pregnancy%20in%20South%20Africa%20Final%2010%20May%202013.pdf. Accessed June 2019.

[99] Lillian P, Mumbango T (2015). Statistical modelling of adolescent pregnancy in Namibia. J Nurs Care.

[100] Salvi F (2018). In the making: constructing in-school pregnancy in Mozambique. Gend Educ.

[101] Gebreselassie T, Shapiro D. Education and Fertility in Sub-Saharan Africa: What Do We Really Know. ICF International; 2016. Available from: https://paa.confex.com/paa/2016/mediafile/ExtendedAbstract/Paper1628/Education%20and%20Fertility%20in%20SSA-March%202016.pdf. Accessed June 2019.

[102] UNAIDS. ESWATINI Country factsheets. HIV and AIDS Estimates. UNAIDS; 2018. Available from: http://www.unaids.org/en/regionscountries/countries/swaziland. Accessed June 2019.

[103] Speizer IS, White JS (2008). The unintended consequences of intended pregnancies: youth, condom use, and HIV transmission in Mozambique. AIDS Educ Prev.

[104] Neal S, Matthews Z, Frost M, Fogstad H, Camacho AV, Laski L (2012). Childbearing in adolescents aged 12-15 years in low resource countries: a neglected issue. New estimates from demographic and household surveys in 42 countries. Acta Obstet Gynecol Scand.

[105] Musekiwe A. Meta-Analysis of Longitudinal Studies with Multiple Effect Sizes. South Africa: University of KwaZulu-Natal Pietermaritzburg; 2017.

